# Genome-wide analysis of KIX gene family for organ size regulation in soybean (*Glycine max* L.)

**DOI:** 10.3389/fpls.2023.1252016

**Published:** 2023-09-27

**Authors:** Gyu Tae Park, Jung-Kyung Moon, Sewon Park, Soo-Kwon Park, JeongHo Baek, Mi-Suk Seo

**Affiliations:** ^1^ Crop Foundation Research Division, National Institute of Crop Sciences, Rural Development Administration (RDA), Wanju-gun, Republic of Korea; ^2^ Gene Engineering Division, National Institute of Agricultural Science, Rural Development Administration (RDA), Jeonju, Republic of Korea

**Keywords:** kix domain, glycine max, haplotype, yield, soybean core collection

## Abstract

The KIX domain, conserved among various nuclear and co-activator factors, acts as a binding site that interacts with other transcriptional activators and co-activators, playing a crucial role in gene expression regulation. In plants, the KIX domain is involved in plant hormone signaling, stress response regulation, cell cycle control, and differentiation, indicating its potential relevance to crop productivity. This study aims to identify and characterize KIX domains within the soybean (*Glycine max* L.) genome to predict their potential role in improving crop productivity. The conservation and evolutionary history of the KIX domains were explored in 59 plant species, confirming the presence of the KIX domains in diverse plants. Specifically, 13 KIX domains were identified within the soybean genome and classified into four main groups, namely *GmKIX8/9*, *GmMED15*, *GmHAC*, and *GmRECQL*, through sequence alignment, structural analysis, and phylogenetic tree construction. Association analysis was performed between KIX domain haplotypes and soybean seed-related agronomic traits using re-sequencing data from a core collection of 422 accessions. The results revealed correlations between SNP variations observed in *GmKIX8-3* and *GmMED15-4* and soybean seed phenotypic traits. Additionally, transcriptome analysis confirmed significant expression of the KIX domains during the early stages of soybean seed development. This study provides the first characterization of the structural, expression, genomic haplotype, and molecular features of the KIX domain in soybean, offering a foundation for functional analysis of the KIX domain in soybean and other plants.

## 1 Introduction

Meeting the increasing demand for future food, feed, and bioenergy requires a significant increase in the production of major crops (Tilman et al., 2011). Soybeans, a major crop, are renowned for their abundant protein and oil content, making them a globally recognized resource for feed and food production and a raw material for biodiesel (Koçar and Civaş, 2013). Hence, increasing soybean yield is a critical issue that must be addressed globally. Efforts to enhance crop yield have ranged from traditional breeding methods to the current digital breeding, with various research being conducted. The viability of such endeavors hinges on the three primary factors influencing crop yield: farming environment, cultivation techniques, and heritability (Scheiner and Lyman, 1989). Among these, heritability is believed to account for more than 50% of the variation in plant characteristics. Plants display a variety of forms and sizes due to genetic factors, with certain traits maintaining consistency within specific species or cultivated varieties. These traits largely determine the size and shape of plant organs by regulating cell division and expansion. These processes are stringently controlled by genetic factors, enabling plants to achieve the desired shape and yield (Krizek, 2009). Despite the importance of such regulation, the precise mechanism governing plant organ size remains inadequately understood, marking this as an intriguing and vital research topic (Wolpert et al., 2015).

In multicellular organisms, organ size determination is regulated through two major pathways: the target of rapamycin (TOR) pathway, which regulates cell growth, and the Hippo pathway, which regulates cell growth, division, and apoptosis (Pan et al., 2004; Horiguchi et al., 2006; Pan, 2007; Tumaneng et al., 2012; Yano et al., 2017). In animals, cell death has been shown to play a role in organ formation, while in plants, organ development depends on cell division and expansion (Mizukami, 2001; Anastasiou and Lenhard, 2007; Krizek, 2009). Moreover, animal organ size regulatory factors do not have plant homologs (Wu et al., 2003; Huang et al., 2005). Instead, plant organ size is controlled by mechanisms such as *BIG BROTHER* (*BB/EOD1*), *DA1*, *ENHANCER OF DA1-1* (*EOD3*), *SUPPRESSOR OF DA1-1* (*SOD7*), *PEAPODs* (*PPD1/*2), and *SAMBA* (White, 2006; Li et al., 2008; Eloy et al., 2012; Li and Li, 2016; Naito et al., 2017; Li et al., 2019b). Furthermore, cell division and expansion are key factors determining final organ size, and the number of cells plays an important role. This suggests that novel mechanisms control plant organ size (Tsukaya and Beemster, 2006). Several key factors have been identified that affect leaf size by regulating the rate and duration of cell division or cell expansion, such as *PEAPOD* (*PPD*) *1* and *PPD2*, which limit the proliferation of meristemoid cells (White, 2006). The *PPD1* and *PPD2* genes encode two transcriptional regulators specific to plants. Knockout or down-regulation of *PPD* genes leads to the formation of large, dome-shaped leaves due to the prolonged proliferation of meristematic tissues (White, 2006; Gonzalez et al., 2015). Recent studies have shown that kinase-inducible domain interacting (KIX) 8 and KIX9 act as molecular bridges between the PPD repressor and the TOPLESS (TPL) co-repressor proteins (Gonzalez et al., 2015; Swinnen et al., 2022). Thus, PPD, KIX, and TPL can form inhibitory complexes that regulate meristemoid proliferation and leaf growth (Gonzalez et al., 2015).

The KIX domains (KIX domain-containing protein) are molecular recognition sites that facilitate protein-protein interactions involved in gene regulation and are conserved across a wide range of organisms, from yeast to plants and animals (Parker et al., 1996; Thakur et al., 2008; Brzovic et al., 2011; Dyson and Wright, 2016). Structurally, KIX domains are characterized by a small protein fold consisting of three helices, named α1, α2, and α3, which form a hydrophobic core and a molecular recognition surface (Radhakrishnan et al., 1997; Zor et al., 2004). The KIX domain surface is designed to accommodate the binding of specific transcription factors, playing a crucial role in regulating gene expression through various interactions. (De Guzman et al., 2006; Thakur et al., 2014). KIX8 and KIX9 have been primarily studied in plants. Notably, mutations or gene knockouts of these genes in soybean, Pisum sativum, and Solanum lycopersicum have been reported to cause an increase in seed and organ size.(Swinnen et al., 2020; Nguyen et al., 2021; Swinnen et al., 2022) (Baekelandt et al., 2018; Li et al., 2018a; Liu et al., 2020; Nguyen et al., 2021; Swinnen et al., 2022). Additionally, it has been demonstrated that single nucleotide polymorphisms (SNPs) in the KIX gene sequence of the *OsMED15* gene are involved in variations in seed production in rice (Li et al., 2019a).

To date, no comprehensive genome-wide investigation and characterization of KIX domains in soybean have been conducted. In our study, we identified KIX domains within the soybean genome and conducted haplotype analysis on them using re-sequencing data from the Korean soybean core collection. This enabled us to explore their potential association with productivity-related traits. Additionally, we investigated the expression patterns of these genes throughout various stages of seed development via transcriptome analyses. Our study aims to provide insights into the role of the KIX domain in regulating plant size, its potential impact on crop productivity, and the molecular mechanisms underlying size regulation. We anticipate that these findings will significantly contribute to crop improvement strategies and efforts to increase harvest yields.

## 2 Materials and methods

### 2.1 Identification of KIX domains in soybean and 58 plant species

**Figure 1:**
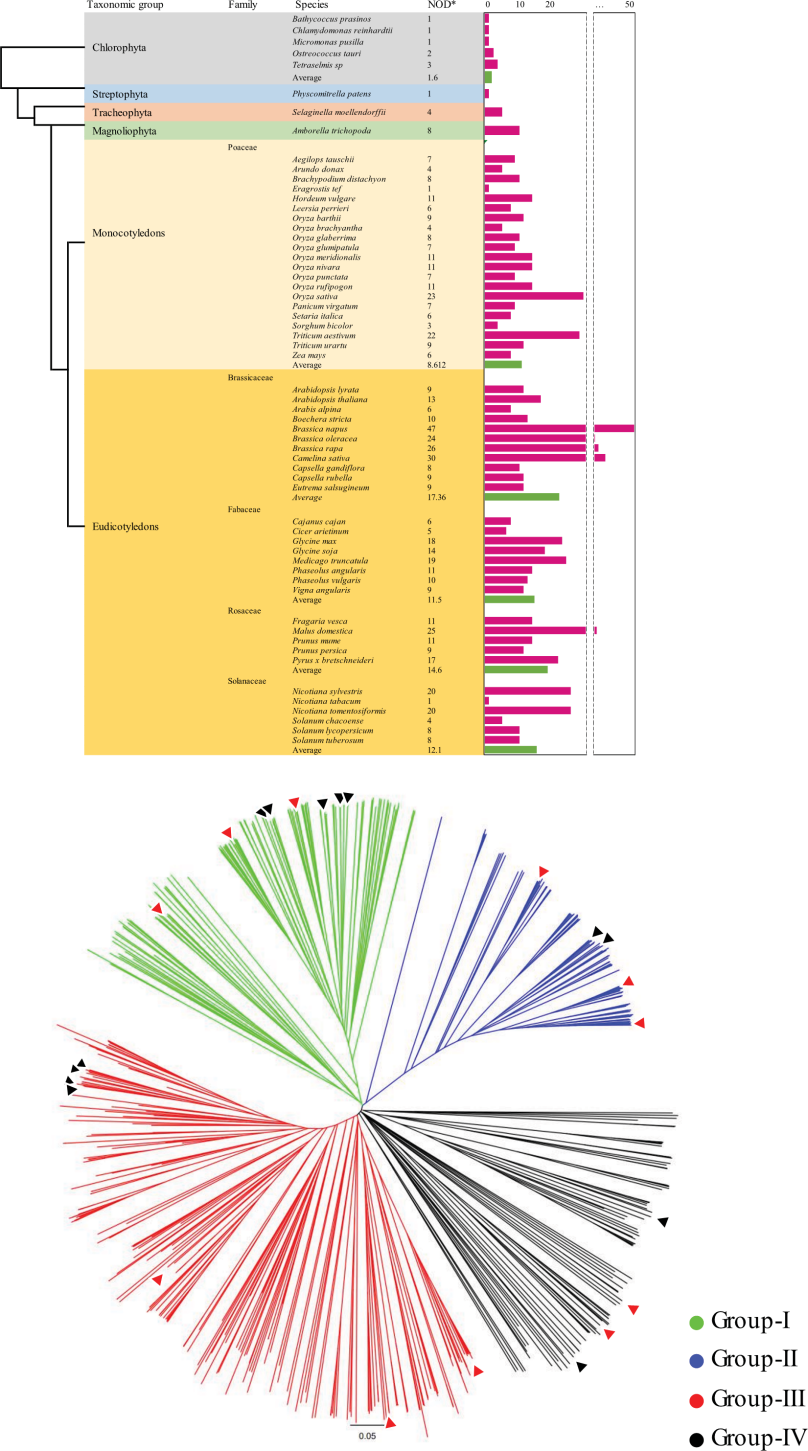
Distribution of KIX domains and phylogenetic relationship among 59 plant species. **(A)** Distribution of KIX domains based on plant classification. The pink bars represent the number of KIX domains present in each plant species. The green bars indicate the average number of KIX domains for each plant group. **(B)** Phylogenetic tree illustrating the protein sequence relationships of 591 identified KIX domains from 59 plant species. The phylogenetic tree was constructed using the neighbor-joining (NJ) method implemented in MEGA-X. The black triangle represents the KIX domain of soybean, while the red triangle represents the KIX domain of Arabidopsis. *NOD, Number of KIX domain.

To find the KIX domain in 59 species (**Figure 1**), KIXBASE (http://www.nipgr.res.in/kixbase/home.php) database was used (Yadav et al., 2017). To identify the KIX domain in soybean (*Glycine max* Wm82.a2.v1 genome version), we used the sequences of Arabidopsis KIX domains as query sequences for performing BLASTP searches on the National Center for Biotechnology Information (NCBI, https://www.ncbi.nlm.nih.gov/), Phytozome website (https://phytozome.jgi.doe.gov/pz/portal.html), and SoyBase databases (https://soybase.org). To confirm the KIX domain in the selected *GmKIX* proteins, we used the KIX_prediction tool in KIXBASE and the Searching Protein Sequence Motifs (MOTIF, https://www.genome.jp/tools/motif/) for validation.

### 2.2 Phylogenetic analysis of KIX domains

Phylogenetic analysis was performed to identify the evolutionary and functional relationship among the species. To better understand the evolutionary relationship among the KIX domains in the genome of various plants, we constructed a phylogenetic tree using the amino acid full-length sequence of 591 KIX domains from 59 species representing major plant groups (**Figure 1A**). The phylogenetic tree was constructed using molecular genetics analysis (Wickett et al., 2014) X with the neighbor-joining (NJ) method and bootstrap analysis was conducted using 1,000 replicates. The consensus tree and unrooted tree were redrawn using Figtree (http://tree.bio.ed.ac.uk/software/figtree/).

### 2.3 Sequence and structure analysis of KIX domains

To predict the function and investigate the structural characteristics of KIX domains, we collected protein and nucleotide sequences of 48 KIX domains from Arabidopsis (11), *soybean* (13), *Cicer arietinum* (5), *Medicago truncatula* (11), and *Phaseolus vulgar* (8) through Phytozome. The sequence information was obtained from Phytozome for *Arabidopsis thaliana* TAIR10, *Cicer arietinum* v1.0, *Medicago truncatula* Mt4.0v1, and *Phaseolus vulgaris* v2.1.The exon/intron structures of the collected KIX genes were visualized using Gene Structure Display Server (GSDS, http://gsds.gao-lab.org/) (Guo et al., 2007). The functional domains of all protein sequences encoded by the candidate KIX genes were predicted using Simple Modular Architecture Research Tool (SMART, http://smart.embl-heidelberg.de/) and Searching Protein Sequence Motifs (MOTIF, https://www.genome.jp/tools/motif/). To confirm the consistency of the KIX domains between Arabidopsis and soybean, Weblogo (https://weblogo.berkeley.edu/logo.cgi) (Crooks et al., 2004) and MEGA-X (Kumar et al., 2018a) were used.

### 2.4 Haplotype analysis of *GmKIX* and phenotypic data collection

Haplotype analyses of *GmKIX* genes were performed using whole-genome re-sequencing data from a soybean core collection of 422 accessions(Kim et al., 2021) (**Supplementary Table 8**). The whole-genome re-sequencing data were utilized using the SRA accession: PRJNA555366, which has been made publicly available by Kim et al., 2021. To filter the re-sequencing data, monomorphic and low-coverage site SNP markers were removed, and those with a minor allele frequency (MAF) less than 0.05 were excluded to minimize the potential influence of rare alleles on the analysis. Additionally, SNPs with missing data for more than 10% of the accessions were removed to reduce the impact of incomplete genotyping information. These filtering steps and genetic admixture analysis were performed using the QTLmax 3.0 program (https://www.qtlmax.com). The soybean core collection was cultivated in an experimental field at the National Institute of Crop Science in 2017 and 2018. After the soybean seeds were harvested and naturally dried to achieve a stable seed weight, phenotypic measurements were conducted indoors. The measured seed agronomic traits were 100-seed weight (100-SW), area, thickness, and major and minor axes (**Supplementary Table 8**).

### 2.5 Population structure and haplotype network analysis

We performed filtering of the soybean core collection re-sequencing data using QTLmaxV3.0. The filtering process included a MAF threshold of < 5%, a limit of 0.1% for missing SNPs, and a stringent threshold for Hardy-Weinberg equilibrium (P-value < 10e-6). Using the filtered set of high-quality SNPs (542,422), we conducted a population structure analysis on the core soybean group, incrementally increasing the K value from 2 to 5, in order to identify an appropriate cluster.

To examine the soybean core collection distribution classified by population structure in haplotypes, we generated a haplotype network using PopART v1.7 (Leigh and Bryant, 2015).

### 2.6 Expression of *GmKIX* genes during seed development stages in soybean

Four cultivars of soybean, namely Hoseo, PI86490, KLS88035, and Soheung-2, were grown in greenhouses to analyze the expression of the *GmKIX* gene during seed development. To analyze the RNA expression levels during seed development, the process was divided into three stages. Stage 1 (S1) included small-seeded cultivars Hoseo and PI86490 with seed sizes less than 3 mm and large-seeded cultivars KLS88035 and Soheung2 with sizes less than 5 mm. Stage 2 (S2) included small-seeded cultivars with seed sizes ranging from 3 to 6 mm and large-seeded cultivars ranging from 5 to 10 mm. Stage 3 (S3) included small-seeded cultivars with sizes greater than 6 mm and large-seeded cultivars with sizes greater than 10 mm (**Supplementary Figure 2**). For each developmental stage, a sufficient number of seeds were promptly collected, immediately frozen in liquid nitrogen, and stored at -80°C for subsequent analysis. Total RNA was extracted from organoid cells using the RNeasy^®^ Plant Mini Kit (Qiagen), and RNA-seq libraries were prepared according to the manufacturer’s protocol. Paired-end RNA-seq reads were generated on the Illumina Genome Analyzer platform, and the quality of the trimmed reads was assessed using FastQC. The expression levels were determined by calculating the reads per kilo-base of the exon per million mapped reads (RPKM). The gene expression profiles were visualized using Pheatmap software (Kolde, 2012). The RNA-seq data reported in this article has been deposited in NCBI under SRA accession: PRJNA1003551.

### 2.7 Statistical analysis

Statistical analyses were conducted using R package. Mean differences among the genotypic groups were analyzed using Fisher’s least significant difference test at a *p* value of 0.05 using PROC GLM.

## 3 Results

### 3.1 Identification and classification of KIX domain in plants

To understand the distribution and evolutionary relationship of the KIX domain in various plant species, we extracted and analyzed amino acid full-length sequence of 591 KIX domains from 59 plant species, including primitive plants, non-seed plants, monocotyledons, and dicotyledons (**Figure 1A** and **Supplementary Table 1**). In primitive plant groups classified as Chlorophyta and Streptophyta, a small number of (1 to 3) KIX domains were identified. *Selaginella moellendorffii*, a more evolved species belonging to Tracheophyta, is considered to have a closer relationship with higher plants among non-seed plants (Banks et al., 2011). In *Selaginella moellendorffii*, four KIX domains were found, whereas eight were detected in *Amborella trichopoda*, currently known as the most basal angiosperm (Islam et al., 2012). With an average of 8.6 KIX domains identified in monocots and approximately 14 in dicots, it was observed that KIX domain-containing proteins were conserved and increased in number throughout the evolutionary process. In monocotyledonous plants, the highest number of KIX domains were identified in *Oryza rufipogon* and *Oryza sativa*. Following them, *Oryza meridionalis* and *Oryza nivara*, closely related to *Oryza sativa*’s ancestors, also showed 11 KIX domains each. Similarly, barley and wheat, two major staple crops, encoded 11 KIX domains each. The abundance of KIX domains identified in plants primarily used as human food or subjected to domestication is intriguing. In dicotyledonous plants, particularly in the genus Brassica, a significant number of KIX domains were identified. *Brassica oleracea* and *Brassica rapa*, diploid species, displayed 24 and 26 KIX domains, respectively. In the tetraploid species *Brassica napus*, approximately twice the number of KIX domains, around 47, were identified. In tetraploid *Brassica napus*, there was a significant increase in the number of KIX domains, whereas in the hexaploid monocot *Triticum aestivum*, a dicotyledonous plant, only 11 KIX domains were identified, making it difficult to determine the variation of KIX domains based on ploidy. Furthermore, no specific trend has been observed concerning chromosome numbers. The interpretation of these results should consider the scope and accuracy of chromosome deciphering in plants while also being mindful of potential biases.

A phylogenetic tree was constructed using the full-length sequences of 591 KIX domains identified from 59 different plant species to investigate their evolutionary relationships and distribution. The phylogenetic analysis classified the proteins into four main groups (**Figure 1B** and **Supplementary Table 1**). Previously identified KIX domains, including KIX8/9, HAC, MED15, and RECQL5, were distinctly divided into these four clusters. The groups were designated as Group-I, Group-II, Group-III, and Group-IV, containing KIX8/9, HAC, MED15, and RECQL5 proteins, respectively. Group-IV, which included RECQL5, also contains WPP proteins and uncharacterized proteins. Therefore, Group-IV was further divided into Group-IV-A, which included RECQL and tryptophan-proline-proline (WPP), and Group-IV-B and Group-IV-C, which include uncharacterized proteins (**Supplementary Table 1**). Group-I contained AtKIX8/9, which interestingly showed even distribution in diverse plants but was rarely detected in mosses and plants in the Poaceae family (**Figure 1B**). Our phylogenetic analysis suggests that, similar to previous studies (Thakur et al., 2013), KIX8/9 proteins belonging to Group-I have evolved from a common ancestral sequence with the KIX domain of HAC proteins classified into Group-II. Interestingly, HAC proteins were detected in all of the 59 plant species except seven. Group-III was classified based on the MED15-related proteins, which means that the proteins belonging to the AtMED15 family were assigned to this group. In the case of the MED15 family, a limited distribution was observed in green algae, while a relatively even distribution was found in monocots and dicots. Approximately 40% of the 591 KIX domains belonged to the MED15 family. This highlights the significance of the MED15 family, as they make up a significant proportion of the KIX domain. Group-IV, a distinct cluster, primarily consisted of RECQL, tryptophan-proline-proline (WPP), and uncharacterized proteins. An interesting aspect of Group-IV was that it primarily comprised plant proteins with little to no research or functional predictions. Group-IV could be further subdivided into Group-IV-A, consisting of RECQL and WPP proteins, Group-IV-B, consisting of uncharacterized proteins closely clustered with MED15, and Group-IV-C, consisting of a total of 52 uncharacterized proteins primarily found in Poaceae plants.

### 3.2 Prediction of KIX domains in *Fabaceae*

**Figure 2:**
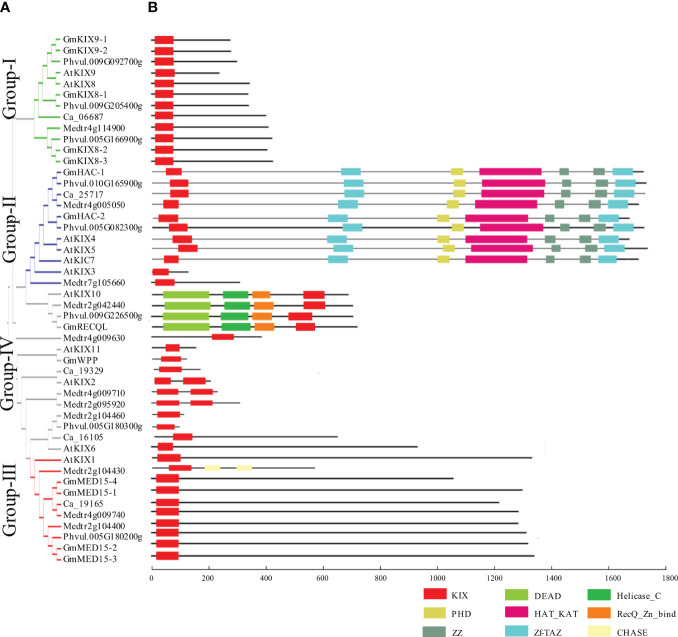
Phylogenetic relationships and structure of KIX proteins from Fabaceae and Arabidopsis. **(A)** Phylogenetic tree of KIX protein amino acid sequences from Arabidopsis, soybean, and three species of Fabaceae. Protein sequences include AtKIX (Arabidopsis), GmKIX (*Glycine max*), Ca (*Cicer arietinum*), Phvul (*Phaseolus vulgaris*), and Medtr (*Medicago truncatula*). The phylogenetic tree was generated using MEGA-X with the neighbor-joining (NJ) method. **(B)**. A schematic diagram of the motifs present in proteins that include the KIX domain. The Red box indicates KIX domain region. Other major domains are described at the bottom of the figure.

Using publicly available complete genome sequences of soybean and Arabidopsis, we identified all possible genes that encode the KIX domain. Ultimately, we identified 13 orthologs corresponding to 11 KIX domains of Arabidopsis (**Figure 2** and **Table 1**). KIX domains from soybean were found to contain one or two KIX domains. To increase the reliability of our results and analyze the trends, we conducted a comprehensive analysis by including 24 KIX domains identified in *Phaseolus vulgaris*, *Cicer arietinum*, and *Medicago truncatula*, in addition to soybean and Arabidopsis. This analysis revealed the presence of four conserved groups (**Figure 2**). Importantly, these four groups corresponded to the divisions made using 591 KIX domain sequences obtained from 59 different plant species, further supporting our findings. Group-I contained *AtKIX8* and *AtKIX9*, which show high similarity to *GmKIX8-1*, *GmKIX8-2*, *GmKIX8-3*, *GmKIX9-1*, and *GmKIX9-2*. The differentiation between *GmKIX8* and *GmKIX9* was based on the similarity to *AtKIX8* and *AtKIX9*, respectively (**Supplementary Table 2**). Group-II contained *AtKIX3*, *AtKIX4*, *AtKIX5*, and *AtKIX7*, reported as p300/CBP related gene, *AtHAC12*, *AtHAC1*, and *AtHAC5* respectively. The two *GmKIX* genes clustered with HAC were named *GmHAC-1* and *GmHAC-2*, respectively. Group-III contained *AtKIX1*, *AtKIX2*, and *AtKIX6*, reported as *AtMED15-1*, MED15-like protein, and *AtMED15-2*, respectively. In soybean, four highly similar genes were identified and named *GmMED15-1*, *GmMED15-2*, *GmMED15-3*, and *GmMED15-4*. In Group-IV, *AtKIX10* and *AtKIX11* were classified and reported as *AtRECQL3* and *AtWPP1*, respectively. In soybean, one similar protein each for *AtRECQL3* and *AtWPP1* was selected and named *GmRECQL* and *GmWPP*, respectively. In three different legume plants, a set of genes corresponding to each group were found to be distributed, with the presence of KIX domains.

**Table 1:** *KIX* gene family and basic properties in soybean and Arabidopsis.

Gene name	Gene Loci	Coordinates	CDS (bp)	Amino acid (aa)	Description	Arabidopsis Orthologues
*GmKIX8-1*	*Glyma.17g112800*	8,907,367-8,911,218	1,218	405	Coactivator CBP, KIX domain	*At3g24150* (*AtKIX8*)
*GmKIX8-2*	*Glyma.13g158300*	27,344,675-27,347,024	1,212	403	Coactivator CBP, KIX domain	*At3g24150* (*AtKIX8*)
*GmKIX8-3*	*Glyma.06g220900*	26,608,060-26,611,018	1,014	337	Coactivator CBP, KIX domain	*At3g24150* (*AtKIX8*)
*GmKIX9-1*	*Glyma.04g066500*	5,539,993-5,542,964	828	275	Coactivator CBP, KIX domain	*At4g32295* (*AtKIX9*)
*GmKIX9-2*	*Glyma.06g067900*	5,191,662-5,195,738	834	277	Coactivator CBP, KIX domain	*At4g32295* (*AtKIX9*)
*GmHAC-1*	*Glyma.08g226700*	18,405,334-18,420,828	5,181	1,727	Histone acetyltransferase Rtt109/CBP Zinc finger,	*At1g16710* (*AtKIX4*)
*GmHAC-2*	*Glyma.15g000300*	23,079-48,897	5,022	1,674	Histone acetyltransferase Rtt109/CBP Zinc finger,	*At1g16710* (*AtKIX4*)
*GmMED15-1*	*Glyma.08g214900*	17,395,957-17,409,920	3,915	1,305	regulation of transcription, DNA-templated transcription cofactor activity	*At1g15780* (*AtKIX1*)
*GmMED15-2*	*Glyma.13g367700*	45,321,490-45,332,706	3,975	1,325	Coactivator CBP, KIX domain	*At1g15780* (*AtKIX1*)
*GmMED15-3*	*Glyma.15g005500*	479,322-491,224	4,041	1,347	Coactivator CBP, KIX domain	*At1g15780* (*AtKIX1*)
*GmMED15-4*	*Glyma.07g027800*	2,201,136-2,220,876	3,189	1,063	regulation of transcription, DNA-templated transcription cofactor activity	*At1g15780* (*AtKIX1*)
*GmRECQL*	*Glyma.09G070600*	7,199,106-7,212,553	2,235	745	TP-DEPENDENT DNA HELICASE Q-LIKE 3	*At4g35740* (*AtKIX10*)
*GmWPP*	*Glyma.14G086900*	7,771,400-7,773,151	372	124	WPP DOMAIN-CONTAINING PROTEIN 1-RELATED	*At5g43070* (*AtKIX11*)

### 3.3 Phylogenetic and structural analysis of *GmKIX* genes

To understand the structural and functional diversity of the KIX domains, a comparative analysis of protein architecture was performed (**Figure 2B**). Additionally, 24 KIX domains from Fabaceae species, including *Phaseolus vulgaris*, *Cicer arietinum*, and *Medicago truncatula*, were included in the study [62]. Using the KIX domains of Arabidopsis as a reference, other similar proteins were clustered into four groups (**Figure 2B**). We observed a remarkable similarity in the KIX domain structures within each group, including the presence and location of the KIX domains, as well as the protein size and arrangement of other domains. The KIX8/9 and MED15 proteins were clustered in Group-I and Group-III, respectively. Apart from two CHASE (Cyclases/Histidine kinases Associated Sensory Extracellular) domains identified in *Medtr2g104430*, no other known domains beyond the KIX domains were detected in these two groups. However, Group-II consisted of HAC proteins, which had conserved domains, including ZF-TAZ, PHD, Hat_Kat11, and ZZ, with the KIX domain. A notable feature in Group-IV is the conservation of RECQL proteins. Distinct from other KIX domains, RECQL proteins preserve the KIX domain at the C-terminal. Additionally, they contain helicase and DEAD domains. The WPP and uncharacterized proteins in Group-IV exhibit diverse sizes and structures, indicating that they are not uniform.

In order to determine the numbers and positions of exon/intron within each *GmKIX* gene, we compared the full-length gDNA sequences with the corresponding Arabidopsis *KIX* gene sequences (**Supplementary Figure 3**). *KIX* genes possess multiple exons and introns, yet their structure and arrangement were largely conserved within the group, typically exhibiting similar patterns. The length of each exon is described in detail in **Supplementary Tables 3, 4**. The genes of Group-I (KIX8/9) had four exons and each exon had a similar length. The *KIX* genes of Group-II (HAC) had the most exons with 16 to 18. Group-III (MED15) showed 11 to 12 exons Furthermore, it was confirmed that RECQL, which was included in Group-IV, preserved 19 exons. The observed exon lengths of *GmKIX* genes were very similar to those reported in Arabidopsis and Rice (Thakur et al., 2013). Additionally, we confirmed that the structure of *KIX* genes in four other Fabaceae species was also similar to that of Arabidopsis, indicating their conservation. On the other hand, it was observed that the UTRs and introns of *KIX* genes exhibited significant variations in length. Specifically, *GmMED15-2* possessed a third intron of approximately 13 kb, whereas the remaining genes within the same group had introns with sizes up to 3 kb. The third intron of *KIX*8/9 also exhibited significant variation, with the third intron of *AtKIX* being 79 bp in length, while *GmKIX9-2* had a much larger intron size of 2,301 bp. In summary, there was a tendency of exon conservation among KIX genes based on their respective groups, while introns exhibited significant variations.

### 3.4 Analysis of the variation and conservation of KIX domains

Through gene structure and motif analysis, we had previously identified differences among groups. Further, we sought to understand functional diversity by analyzing the variations and patterns in the amino acid sequence of the primary KIX domains. The KIX domain is characterized by a conserved structural fold consisting of three helix bundles that mediate the interaction with binding proteins. Hydrophobic interactions between helices contribute to the formation of a robust fold in the domain and aid in stabilizing the binding with interacting partners (Radhakrishnan et al., 1997; Brzovic et al., 2011). Despite the conservation of this fold, KIX domain sequences exhibit significant diversity, contributing to their functional flexibility (Yadav et al., 2017). To confirm the preservation of the 3-helix structure in the selected KIX domains from soybean and the diversity of KIX domain sequences, we analyzed the KIX domain sequences of soybean and Arabidopsis (**Supplementary Table 5**, and **Supplementary Figure 2, 4**). The KIX domain of the selected *GmKIX* genes also maintained the 3-helix bundle structure, and the amino acid residues critical for structural stability were more highly conserved than other residues. Additionally, we investigated the conservation of domain sequences within each group of GmKIX proteins. As a result, among the four groups, Group-I, which included KIX8/9, was found to have the highest sequence conservation in the KIX domains. Query coverage scores were above 97%, and the positive scores were approximately 90%, indicating a match with Arabidopsis. Groups containing proteins such as MED15, HATs, and RECQL exhibited positive scores of around 55-62% for the KIX domains, suggesting their potential to contribute to functional flexibility. An interesting observation is the rarity of fully conserved amino acid sequences in the KIX domain sequences between soybean and Arabidopsis. In the domain sequence alignment, only the 22^nd^ and 49^th^ amino acids were perfectly conserved as Arg and Glu, respectively (**Supplementary Figure 4**). Other sequences appeared to have diverged and undergone variations based on their respective genes and groups.

We reconstructed the evolutionary tree using only KIX domain sequences diversified according to their functionalities (**Supplementary Figure 4**). The tree constructed using only KIX domain sequences exhibited remarkably high similarity to that constructed using full amino acid sequences. This result suggests that the sequence variations in the KIX domain have occurred in conjunction with the functional diversification of KIX domain-containing proteins. It is anticipated that the function of proteins containing the KIX domain can be predicted solely based on the sequences. We identified six key amino acid sequences that contribute to the classification of KIX domains into four groups. The selection of these six amino acid sequences was primarily based on their differential conservation across the four groups of KIX domains. In particular, the 66^th^ amino acid exhibited distinct characteristics in each group: Glu, a polar and negatively charged amino acid, in Group-I; Lys, a polar and positively charged amino acid, in Group-II; Gln, a polar and uncharged amino acid, in Group-III; and Gly, an amino acid classified under special cases group, in Group-IV. Hence, the selected six amino acids can serve as benchmarks for differentiating the functions of KIX domains through one or multiple combinations. Therefore, these six amino acids have undergone increased diversification during evolution, allowing for functional diversification of the KIX domain in polyploid plants, including soybean.

### 3.5 Analysis of association between *GmKIX* gene haplotype and seed-related agronomic traits

Gene structure and domain sequence analyses revealed that *GmKIX* genes within the same group exhibit conserved structures and domain sequences. Furthermore, we focused on variations in exon sequences to conduct a detailed analysis of sequence variations within the conserved coding region. In order to investigate the variations and diversity within *GmKIX* genes that have undergone functional differentiation, we performed haplotype analysis using re-sequencing data from the soybean core collection consisting of 422 accessions.

**Figure 3:**
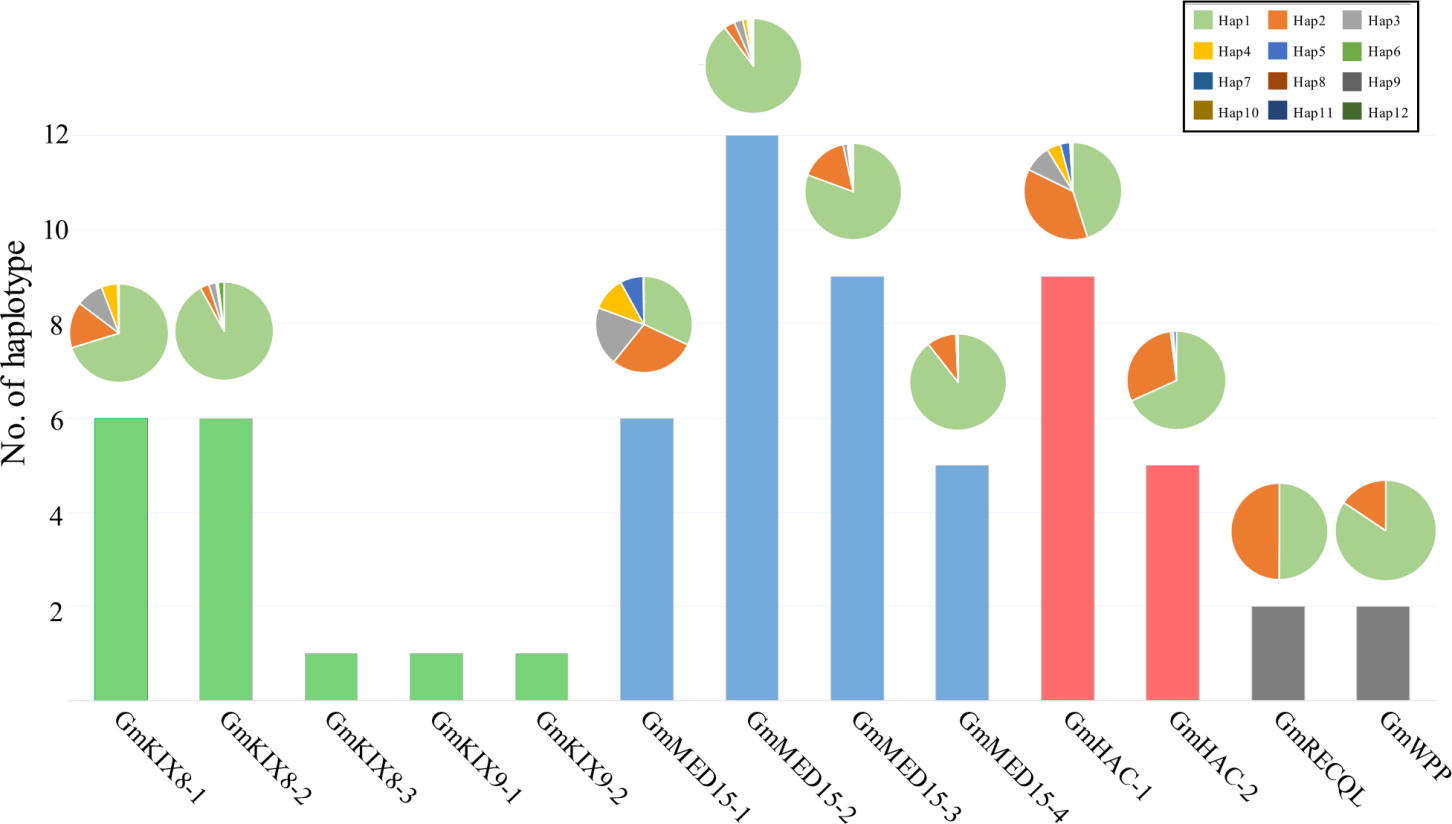
Haplotype analysis for *GmKIX* genes and distribution of haplotype variations across each gene. The bar chart represents the number of haplotypes for each *GmKIX* gene, and the pie chart illustrates the distribution of each haplotype. The box on the right represents the information of the pie chart.

We removed heterozygous variants and sequencing errors from the filtered mutations in the re-sequencing data of 422 soybean varieties. We focused on haplotypes that exhibited non-synonymous substitutions and functional InDels among the filtered mutations. The analysis revealed an average of 3 haplotypes in Group-I, 8 in Group-II, 7 in Group-III, and 2 in Group-IV (**Figure 3**). The haplotype distribution of each *GmKIX* gene was predominantly characterized by a single dominant haplotype, except for *GmMED15-1*, *GmHAC-1*, and *GmRECQL*, which exhibited two or more equally distributed haplotypes. Interestingly, *GmKIX* genes belonging to the same group, namely *GmKIX8-3*, *GmKIX9-1*, and *GmKIX9-2*, have approximately 900 bp exons, and among them, no significant variations were observed in the coding region. On the other hand, *GmKIX8-1* and *GmKIX8-2* harbored a 1,200 bp exon and exhibited six distinct haplotypes. These findings suggest that in cases where variations are limited, such as in *GmKIX8-3*, *GmKIX9-1*, and *GmKIX9-2*, sequence changes in the coding region may potentially have significant implications in the plant system.

**Figure 4:**
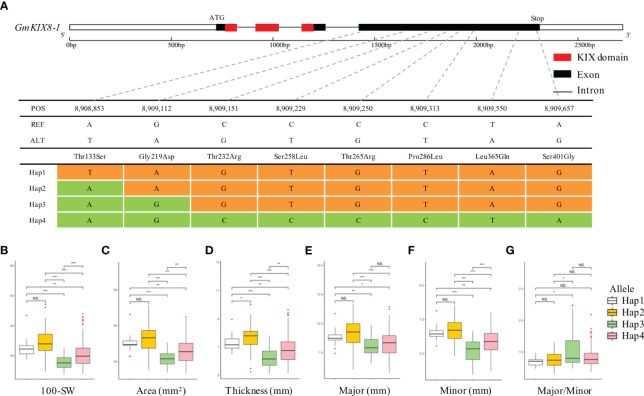
Association between haplotype in *GmKIX8-1* and seed agronomic traits. **(A)** Haplotype analysis of *GmKIX8-1* based on re-sequencing data from the soybean core collection. Four haplotypes of the *GmKIX8-1* gene were determined based on the polymorphisms detected in the coding region. **(B-G)** boxplot displays the distribution of various agronomic traits (100-SW, area, thickness, and minor and major axes) values for the four haplotype types of *GmKIX8-1*. NS, not significant, *p < 0.05, **p < 0.01, ***p < 0.001. 100-SW, 100 seed weight.

**Figure 5:**
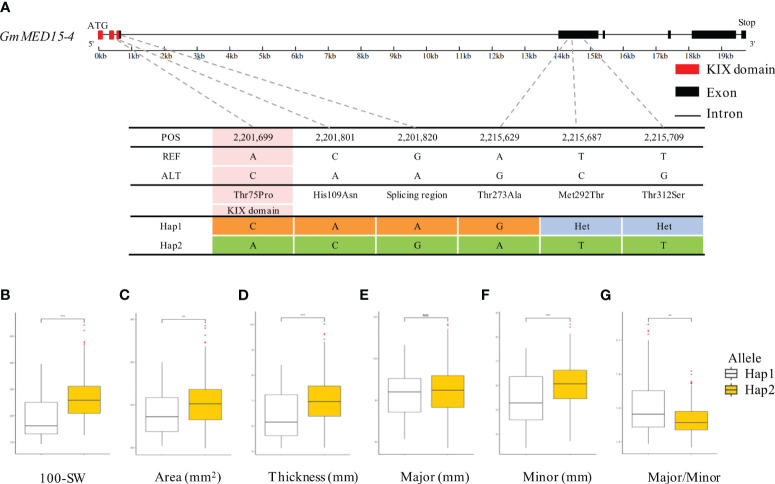
Association between haplotype in *GmMED15-4* and seed agronomic traits. **(A)** Haplotype analysis of *GmMED15-4* based on re-sequencing data from the soybean core collection. Two haplotypes of the *GmMED15-4* gene were determined based on the polymorphisms detected in the coding region. **(B-G)** boxplot displays the distribution of various agronomic traits (100-SW, area, thickness, and minor and major axes) values for the two haplotype types of *GmKIX8-1*. NS, not significant, **p < 0.01, ***p < 0.001. 100-SW, 100 seed weight.

**Figure 6:**
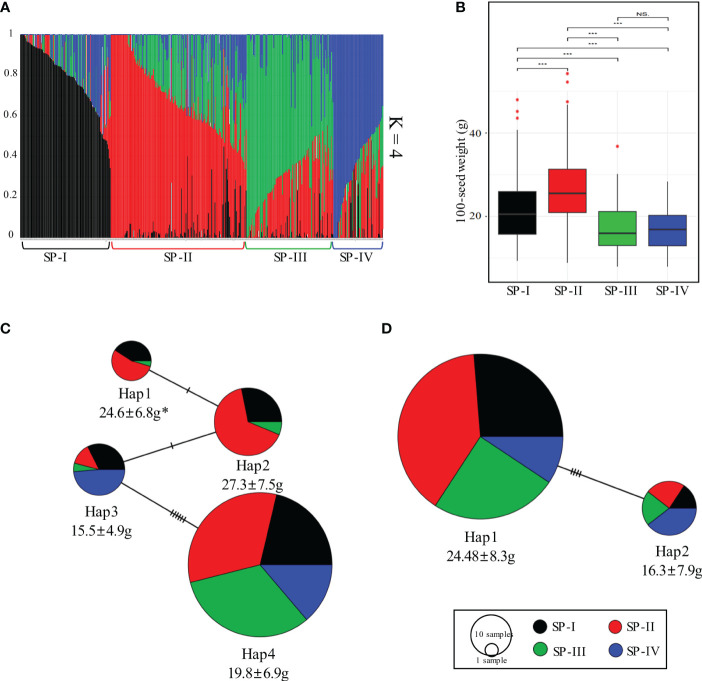
Analysis of population structure in the soybean core collection and haplotype network of *GmKIX8-1* and *GmMED15-4*. **(A)** Population structure of the 422 soybean core collection with 542,422 SNPs. **(B)** Boxplot of 100-seed weights of resources distributed among four subpopulations. **(C)** The haplotype network of *GmKIX8-1*. **(D)** The haplotype network of *GmMED15-4*. * 100-SW (mean ± standard deviation). ***p < 0.001, NS, not significant. 100-SW, 100 seed weight.

We performed the haplotype-based association analysis of the 13 *GmKIX* genes related to seed-related agronomic traits using 422 soybean accessions. As a result, we confirmed the correlation between the genetic variations of *GmKIX8-1* and *GmMED15-4* and the phenotypes in seed-related agronomic traits (**Figures 4**, **5**). *GmKIX8-1*, with four main haplotypes, had a sufficient number of resources for statistical analysis (**Figure 4A**). Variations in the haplotypes of *GmKIX8-1* were observed only in the fourth exon, where a total of 8 non-synonymous mutations were present. Upon examination of seed-related agronomic traits according to each haplotype, Haplotypes Hap-1 and Hap-2 exhibited a relatively larger and heavier seed shape, while Hap-3 and Hap-4 showed a relatively smaller distribution in seed-related agronomic traits. The distribution of 100-SW revealed that the mean values for Hap-1, Hap-2, Hap-3, and Hap-4 were 24.6 ± 6.8 g, 27.3 ± 7.5 g, 15.5 ± 4.9 g, and 19.8 ± 6.9 g, respectively (**Figures 4B**, **6C**). While there was no significant difference between Hap-1 and Hap-2, there was a significant difference between these two haplotypes and the other haplotype groups. The SNP that distinguishes Hap-1 & 2 from Hap-3 & 4 is the G to A change at position 8,909,112, resulting in the conversion of Gly^219^ to Asp^219^, which enables differentiation between the two haplotypes. Based on these results, it can be understood that the one-base substitution at position 8,909,112 may impact the function of *GmKIX8-1* and thus affect the development related to seed-related agronomic traits. SNPs of *GmMED15-4* were distinguished as two haplotypes. Specifically, a variation in the nucleotide sequence at position 2,201,699 of the KIX domain of *GmMED15-4* was observed with A and C alleles, which encoded Thr^75^ and Pro^75^, respectively (**Figure 5A**). The correlation between haplotypes containing the variation in the amino acid sequence at position 75 and seed agronomic traits showed mostly small tendencies in Hap-1 compared to Hap-2. The average 100-SW (**Figure 5B**) for Hap-1 and Hap-2 were 24.48 ± 8.3 g and 16.3 ± 7.9 g, respectively, with a p-value <0.01 indicating a significant difference between the average 100-SW for Hap-1 and Hap-2. The two genes, *GmKIX8-1* and *GmMED15-4*, showed differences not only in 100-SW but also in area, thickness, and minor axis, while no correlation was found between the major axis and haplotype. Both *GmKIX8-1* and *GmMED15-4* have been reported to have an impact on plant size and seed size through genome editing using CRISPR-Cas9 in soybean, as well as significant differences in SNP and seed development and morphology in rice (Thakur et al., 2013; Nguyen et al., 2021). These findings further enhance the credibility of our association analysis between haplotypes and seed-related agronomic traits. No significant difference was observed between the haplotypes of other *GmKIX* genes and seed-related agronomic traits in this study.

We further conducted population structure analysis and investigated the distribution of seed-related traits based on the genetic data of the core soybean collection in Korea. After filtering, a total of 542,422 high-quality SNPs were obtained from the re-sequencing data of the soybean core collection. Population structure analysis was performed using the high-quality SNPs, and based on the reference results of the 180K SNP analysis of the core soybean collection by (Lee et al., 2022), the optimal value for K was determined to be 4 (**Figure 6A**). The resulting clusters were labeled as Subpopulation (SP)-I, SP -II, SP-III, and SP-IV, comprising 105, 158, 101, and 51 resources, respectively. To investigate the association between the four SP and 100-SW, we initially performed linear regression analysis with 100-SW as the dependent variable. In this analysis, we used q-values obtained from the four subpopulations as covariates. Due to the high correlation among the q-values, which raised concerns of multicollinearity, we systematically omitted one q-value at a time and conducted the regression analysis with the remaining three q-values (**Supplementary Table 6**). Consistently, the majority of q-value covariates displayed a statistically significant relationship with 100-SW values (p < 0.01). The R^2^ value was determined as 0.46. Furthermore, in order to assess the association between the candidate gene’s haplotype and 100-SW, we conducted a linear regression analysis with population structure effects as covariates, revealing a statistically significant relationship with p < 0.01 (**Supplementary Table 7**). Upon investigating the distribution of 100-SW among the resources within each cluster, it was observed that SP-II exhibited significantly higher 100-SW values than the resources in the other clusters (**Figure 6B**). Furthermore, significant differences were observed among the clusters regarding the admixture results. We further investigated the relationship between subpopulations within the haplotype distribution of the *GmKIX8-1* and *GmMED15-4* genes and their association with 100-SW (**Figures 6C, D**). In the case of *GmKIX8-1*, among its four haplotypes, Hap-1 and Hap-2 were characterized by relatively larger seed sizes, and these haplotypes predominantly constituted the resources of SP-II, which had the largest average seed sizes. Subsequently, the resources within SP-I were found to be distributed next. In contrast, Hap-3 and Hap-4, characterized by relatively smaller seed sizes, exhibited a higher distribution of SNPs in resources within SP-III and SP-IV, where smaller resources were predominant. Regarding the haplotypes of *GmMED15-4*, Hap-1, which includes relatively larger seeds, the highest number of SNPs in resources was seen within SP-II. On the other hand, Hap-2, characterized by relatively smaller sizes, had the highest proportion of resources classified under SP-IV, consisting of smaller resources. The results of the haplotype network and population structure analysis strengthen the confidence in the association analysis between *GmKIX* gene haplotypes and seed-related agronomic traits using re-sequencing data from the core soybean collection.

### 3.6 Differential expression profile of *GmKIX* genes

**Figure 7:**
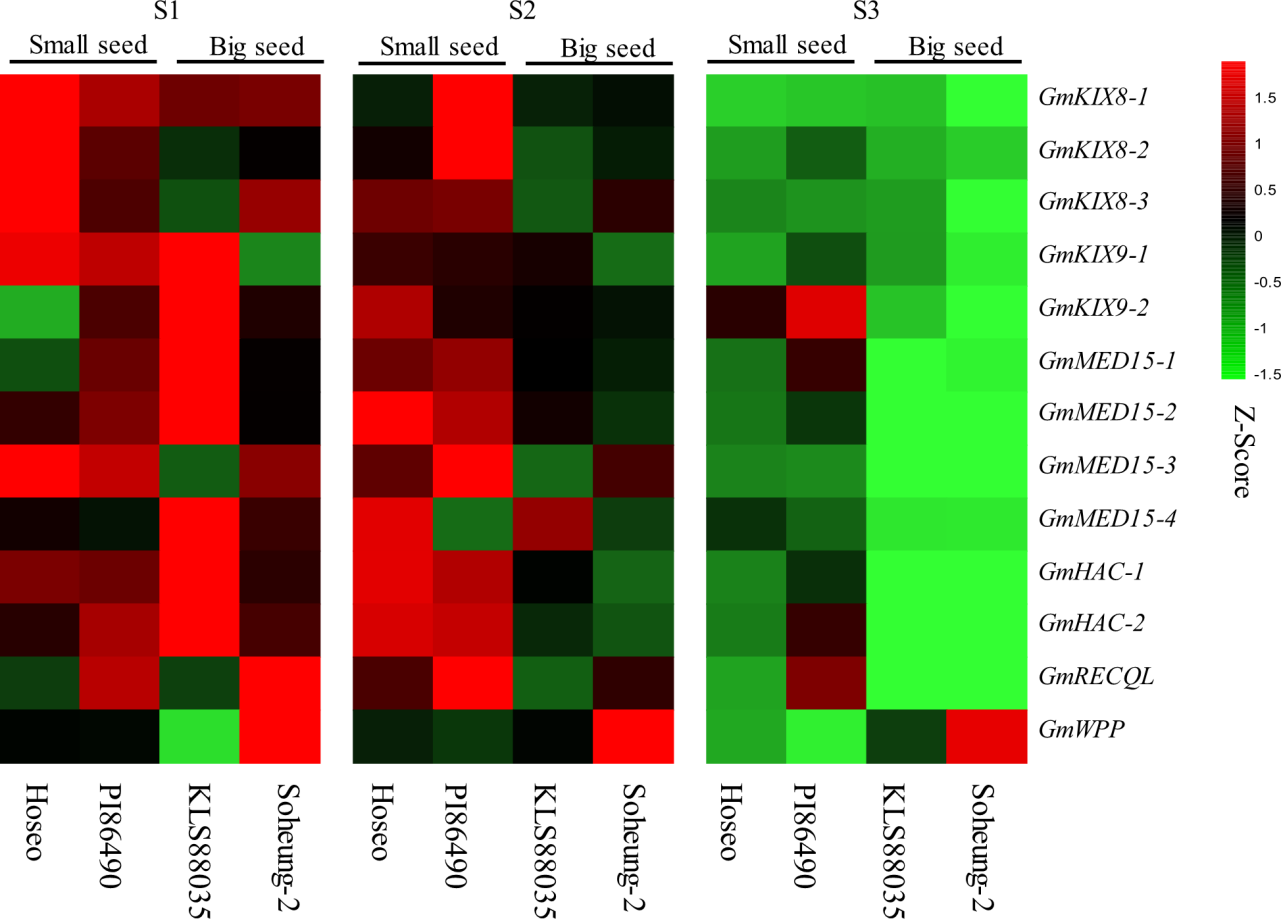
Heatmap of *GmKIX* genes expression in three stage of seed development. Expression analysis of *GmKIX* genes was conducted based on the developmental stages of seeds in four variations: Hoseo, PI86490, KLS88035, and Soheung-2. The RPKM values of each gene were normalized to Z-scores. Detailed information regarding the developmental stages and resources can be found in **Supplementary Figure 2**.

Transcriptome sequencing (RNA-seq) data from three different developmental stages of four soybean accessions were used. RPKM values were standardized as Z-scores to compare the expression according to the seed development stage for each *GmKIX* gene. The expression of *GmKIX* genes was downregulated according to the seed development process and showed differences between small (Hoseo and PI86490) and big seeds (KLS88035 and Soheung-2) (**Figure 7**). Initial seed Stage (S1) had a high expression of *GmKIX* genes in both small and big seed breeds compared to the other stages. There was a difference in the expression of the *GmKIX* gene between the small seed and the big seed in the expansion stage (S2). Most of the *GmKIX* genes still showed relatively high expression in small seeds, while the amount of expression in the big seeds was significantly reduced compared to S1. In the filling stage (Sterner and Berger, 2000), it can be seen that the amount of expression decreased in both small and big seed varieties. Interestingly, it was confirmed that the small and big seeds showed similar expression patterns in S1 and S2, while the expression was maintained in the two accessions with small seeds in the vigorous seed development stage, such as S2. In addition, normalized RPKMs (log_2_ scale) were checked to detect the number of genes actively expressed in the *GmKIX* gene (**Supplementary Figure 5**). The *GmKIX9-1* and *GmKIX9-2* genes showed lower expression than other *GmKIX* genes, and the *GmMED15* and *GmHAC* genes showed relatively higher expression. Upon investigating the expression patterns during the early stages of seed development, the expansion phase, and the seed filling phase, we concluded that the expression of these *GmKIX* genes is involved in the initial stages of seed development and may influence size determination.

## 4 Discussion

Crop yield improvement is one of the most critical topics in plant breeding. A variety of genes and mechanisms are involved in plant development, influencing yield. The KIX domain has primarily been reported in Arabidopsis and is known to influence agronomic traits such as organ development and grain size (Patel et al., 2004; Deng et al., 2007; Thakur et al., 2013; Kumar et al., 2018b; Li et al., 2018a; Röhrig et al., 2018; Liu et al., 2020; Swinnen et al., 2020; Nguyen et al., 2021; Swinnen et al., 2022). However, research on the role of KIX domains in soybeans is limited. The KIX domain possesses unique characteristics that distinguish it from other domains. The KIX domain sequence possesses a scaffold due to its triple helix bundle and structural stabilization (Thakur et al., 2014; Yadav et al., 2017). The typical characteristics of the KIX domain have also been confirmed in soybeans (**Supplementary Figure 4**). However, differences in full-length DNA region, amino acid structure, and domain region are observed depending on their specialized functions (**Figures 2**–**4**) (Yadav et al., 2017). These properties of the KIX domain make detection difficult not only within plants but also across taxa (Thakur et al., 2013; Thakur et al., 2014). Hence, research on KIX domains necessitates further granularity, with a notable lack of such studies in numerous plants.

Firstly, we sought to understand the structural characterization of KIX domains in plants. By systematically investigating KIX domain proteins in 59 plant species ranging from unicellular aquatic algae to terrestrial higher plants, we demonstrated their functional significance and origin. Generally, the number of KIX domains increased as they evolved from their ancestors (**Figure 1A**). Interestingly, KIX domains were found in unicellular aquatic algae, suggesting their ancient origin and functional conservation. KIX domains were detected in 1 to 3 copies in five algal species, while 1 to 47 orthologous proteins were identified in both monocots and dicots, indicating a rapid gene expansion of KIX domain proteins in higher plants. Moreover, the number of KIX domain members in terrestrial plants showed varying degrees of expansion compared to aquatic algae (Li et al., 2018b). Research on the conservation of KIX domains in various plants revealed that KIX domains are conserved in monocots or dicots and have evolved into four major conserved forms (**Figure 1B**). As they evolved multicellularity and became exposed to diverse environments, KIX domains diversified and expanded according to complex mechanisms. Gene duplication and expansion always follow functional diversification. Functional diversification can provide new genes that can adapt to new environments (Trezise and Collin, 2005; Han et al., 2007; Nacher et al., 2010). In plants, the expansion of gene families represents the differentiation of physiological functions of each isoform, regulating aspects of expression sites, and helping the organism adapt to different environmental conditions later on (Plomin, 1986; Rensing, 2014).

Furthermore, we ultimately identified 13 KIX domains in soybean (**Table 1** and **Figure 2**). We constructed a system-generated tree to distinguish duplicated and derived genes and investigate the pattern of KIX domain family expansion during evolution (**Figure 2**). We divided the KIX domains into four clades and inferred their potential functions. Previously, the KIX domain was characterized in CBP, MED15, and RECQL5 helicase (Yadav et al., 2017). However, we propose the addition of KIX8/9 as another major class. When considering the phylogenetic analysis results (**Figures 2A**, **4B**) and the conservation of domain sequence alignment coverage score of over 90% (**Figures 3A**, **4**), it can be concluded that KIX8/9 can be considered an independent group. Therefore, we categorized the KIX domain into four distinct groups and proposed four main functional roles. The clear tree classification of the KIX domain into four distinct groups is an intriguing observation. It is even more surprising that we can observe the diversification of conserved KIX domain sequences among the four distinct protein groups, highlighting the close relationship between the patterns of the KIX domain and protein functions (**Supplementary Figure 4**). While maintaining the characteristic three α-helix structure of the KIX domain, the diversification of binding sites with their respective protein targets has occurred, leading to functional specialization. Based on these findings, it is suggested that the patterns of amino acid sequences can be utilized for further studies.

We have confirmed the potential maintenance of function in KIX domains in soybean through structural and molecular characterization and phylogenetic relationship. We specifically focused on the involvement of these proteins in plant productivity, specifically plant size. To investigate this possibility, we examined the correlation between variations in KIX domains and changes in seed-related agronomic traits. As a result, we observed that variations in the coding region of *GmKIX8-1* and *GmMED15-4* genes were associated with changes in seed size factors (**Figures 6**, **7**). The intriguing discovery is that variations in the coding sequences (coding region) of *GmKIX* genes are associated with various seed-related agronomic traits. To support this hypothesis, we constructed a population structure and validated whether there were differences in seed production-related traits according to the genetic diversity within the core population. The analysis revealed that the four populations generated from the genetic data of the core population exhibited significant differences in seed size (**Figure 6**). This suggests genetic variations within the soybean core population may be involved in regulating seed production. Furthermore, the population we used allowed us to identify factors, including KIX domains, associated with soybean productivity.

As additional evidence, it has been reported in other plants that *AtKIX8/9* and their orthologous genes are involved in seed and organ size (Swinnen et al., 2020; Nguyen et al., 2021; Swinnen et al., 2022). In fact, according to recent studies, *KIX8*/9 participate in organ and seed size by forming complexes composed of KIX/PPD/MYC and PPD/KIX/TPL, thereby regulating protein-protein interactions (White, 2006; Gonzalez et al., 2015; Wang et al., 2016; Baekelandt et al., 2018; Li et al., 2018a; Liu et al., 2020). The knockout of *KIX8*/9 ultimately leads to the suppression of D3 cyclin expression, resulting in controlled cell proliferation, increased cell numbers, and enhanced plant productivity (Li et al., 2018a; Nguyen et al., 2021; Swinnen et al., 2022). Notably, while the overall plant size increased, it did not cause significant growth or physiological issues, leading to yield improvements. Moreover, QTL studies on the 100-SW in soybean have been extensive. Among these, *qSw17* is well-known for its influence on soybean seed weight (Hoeck et al., 2003; Panthee et al., 2005; Liu et al., 2007; Teng et al., 2009; Kim et al., 2010; Liu et al., 2013; Kato et al., 2014; Yan et al., 2017; Liu et al., 2018). It has been reported that the *GmKIX8-1* gene, located within *qSw17*, causes a fast neutron (FN) mutation by losing its function through genome editing, resulting in increased productivity. Not only *KIX8* but also *KIX9* yielded similar results in Arabidopsis by restricting their function (Liu et al., 2020), as well as in tomato (Swinnen et al., 2022). When *KIX8* and *KIX9* were both knocked out, seed size and weight increased significantly (Liu et al., 2020). Based on these findings, it is anticipated that the *KIX8/9* genes present in soybean may also be involved in plant development and cell division, potentially impacting yield enhancement. MED15 in group III is also known to interact with various transcription factors, and considering the association between the discovered SNPs and seed morphology in rice, the variation in the *MED15* gene should also be taken into account (Thakur et al., 2013). MED15 is a subunit of the Mediator complex, essential for transcription regulation in eukaryotes involving RNA polymerase II (Malik and Roeder, 2005). MED15 is involved in various signaling pathways, contributing to cellular survival, differentiation, development, and metabolic regulation (Nakatsubo et al., 2014; Kumar et al., 2018b). Furthermore, MED15 is involved in signaling pathways such as β-catenin and TGF-β, which can influence biological processes like cell division, differentiation, and cell motility (Conaway and Conaway, 2011). Therefore, MED15 is a multifunctional protein with important roles in transcription regulation and cellular processes. Although information on the function of plant MED15 is limited, its role in salicylic acid signaling has been reported in Arabidopsis (Canet et al., 2012), classified based on KIX domains in rice, as reported in previous studies (Thakur et al., 2013). Based on our analysis and previous studies, Group-I and Group-III are more important in enhancing plant productivity among the four groups. Among the four KIX domain clades, *KIX8*/9 and *MED15* exhibited approximately twice the orthology in soybean compared to Arabidopsis. It is speculated that after the soybean duplication event, their functions diversified, playing an increasingly important role in plant growth. These results suggest that *KIX8/9* and *MED15* genes have the highest potential to be involved in plant production. Interestingly, the association study between haplotype and seed-related agronomic traits supported this possibility.

In addition, the HAC and RECQL proteins, which are conserved in soybean, also possess significant potential related to productivity. In the plant KIX domain, HAT-classified proteins and RECQL proteins exhibit a unique characteristic, where they contain various domains apart from the KIX domain, unlike KIX8/9 and MED15 proteins (**Figure 2B**).

RECQL proteins have been reported to interact with RNA polymerase in mammals and play a crucial role in suppressing chromosomal exchange. RECQL5 is a DNA helicase containing a KIX domain and is involved in various DNA metabolic processes, including replication, repair, and double-strand break repair (Peng et al., 2019; Andrs et al., 2020; Ding et al., 2021). In addition to the KIX domain, RECQL5 has a helicase domain responsible for unwinding DNA structures and promoting the response to DNA damage during replication (Bernstein et al., 2010). However, research on their function in plants remains limited. RECQL proteins have been conservatively identified not only in Arabidopsis and soybean but also in *Medicago truncatula* and Fabaceae, suggesting that they may also play an essential role in maintaining genome stability in plants. The presence of these diverse domains has been speculated to result from factors such as functional diversity and evolutionary adaptation. Functional diversity enables HAT and RECQL proteins to perform a wide range of functions, including protein-protein interactions, histone recognition, acetylation reactions, DNA helicase activity, and nucleic acid binding, allowing them to be involved in diverse regulatory mechanisms (Chan and La Thangue, 2001; Kalkhoven, 2004; Hu et al., 2009; Ramamoorthy et al., 2013; Peng et al., 2019). In contrast, the existence of a single KIX domain in KIX8/9 and MED15 proteins indicates a specialized role in specific cellular processes such as transcription regulation or signal transduction. In such cases, although the amino acid length might be larger, additional domains beyond the core functional domain may not be necessary for the protein function. In summary, the presence of multiple domains in HAT and RECQL proteins and a single KIX domain in KIX8/9 and MED15 proteins reflects diversity and evolutionary adaptation. This diversity allows these proteins to participate in a wide range of cellular processes and regulatory mechanisms.

In this study, we identified 13 KIX domains based on 11 Arabidopsis KIX domains. To predict and classify the functions of soybean KIX domains, we employed various approaches including gene structure analysis, domain structure characterization, phylogenetic analysis, comparative transcriptomics, and SNP-based haplotype studies. As a result, soybean domains could be categorized into four groups based on functional divergence and sequence conservation. Furthermore, through haplotype analysis, we confirmed the significance of *GmKIX8-3* and *GmMED15-4* in soybean seed agronomic traits, suggesting their potential contribution to crop yield improvement. Our findings provide a robust foundation for the evolutionary history and molecular characterization of KIX domains, as well as the investigation of mechanisms related to plant productivity.

## Data availability statement

The original contributions presented in the study are included in the article/**Supplementary Material**. Further inquiries can be directed to the corresponding author.

## Author contributions

M-SS conceived and supervised the experiment, and revised the manuscript. GTP analyzed the data and wrote and revised the manuscript. S-KP and J-KM assisted in field research and editing the manuscript. SP assistance in the analysis of genomic data. JHB measured the agronomic traits of soybean seeds using image program. All authors contributed to the article and approved the submitted version.

## Supplementary material

Sequence comparison of the N-terminal KIX domain region with Arabidopsis and soybean.suStages of soybean seed development and sampling times of four soybean varieties. Heatmap of 13 GmKIX domains expression in three stage of seed development.
