# Supplementary material for: Genome-wide analysis of KIX gene family for organ size regulation in soybean (Glycine max L.)
Source: Front Plant Sci. 2023 Sep 27;14:1252016. doi: 10.3389/fpls.2023.1252016 (PMC10565003; doi:10.3389/fpls.2023.1252016)
Supplement: Supplementary file 1 [file DataSheet_1.docx]

Supplementary Material

Genome-wide haplotype analysis and molecular characterization of KIX gene family in soybean (Glycine max L.)

Gyu Tae Park^1^, Jung-Kyung Moon^1^, Sewon Park^1^, Soo-Kwon Park^1^, JeongHo Baek^2^, Mi-Suk Seo^1^*

*** Correspondence:** Mi-Suk Seo: sms1030@korea.kr

**Supplementary Figure 1. Sequence comparison of the N-terminal KIX domain region with Arabidopsis and Soybean.** (A) All KIX domain regions in Arabidopsis and Glycine max. (B), (C), and (D) are compared to the KIX domain sequence corresponding to Group-I, Group-II, and Group-III, respectively. The amino acid sequences of each conserved domain are displayed on the x-axis, and the height of each letter corresponds to the degree of conservation for each residue. The three helix structures present in the KIX domain protein are represented as Helix1, Helix2, and Helix3, respectively. The size of the letters indicates a relatively high degree of conservation. Conserved amino acids that stabilize the structure are marked with black triangles.

**Supplementary Figure 2. Stages of soybean seed development and sampling times of four soybean varieties.** (A) Seed developmental stages in soybean big seed (KLS88035 and Soheung-2) and small seed (Hoseo and PI86490) variants. Bars = 1mm. (B) Table of seed size and weight for four soybean varieties, based on 100-seed weight.

**Supplementary Figure 3. Analysis of the exon/intron structure of KIX domains in soybean, Arabidopsis, and Fabaceae.** The exon/intron structure of each gene group is depicted. Black boxes represent exons, black lines represent introns. (A), (B), (C), and (D) represent the exon/intron structures of KIX8/9, MED15, HAC, and RECQL, respectively, while (E) categorizes genetically diverse genes with heterogeneous structural characteristics.

**Supplementary Figure 4. Multiple sequence alignment and phylogenetic tree of KIX domain sequence from soybean and Arabidopsis.** (A) Alignment of KIX domain sequences from four groups. Blue lines represent the three helical structures. Black arrows indicate conserved amino acid sequences that differentiate each group. The black arrows represent candidate marker sequences capable of distinguishing the four groups. (B) Phylogenetic tree constructed using amino acid sequence of KIX domains. (C) Phylogenetic tree generated using full-length amino acid sequences of KIX domain-containing proteins. Green, blue, red, and black lines in the phylogenetic trees represent Group-I, Group-II, Group-III, and Group-IV, respectively.


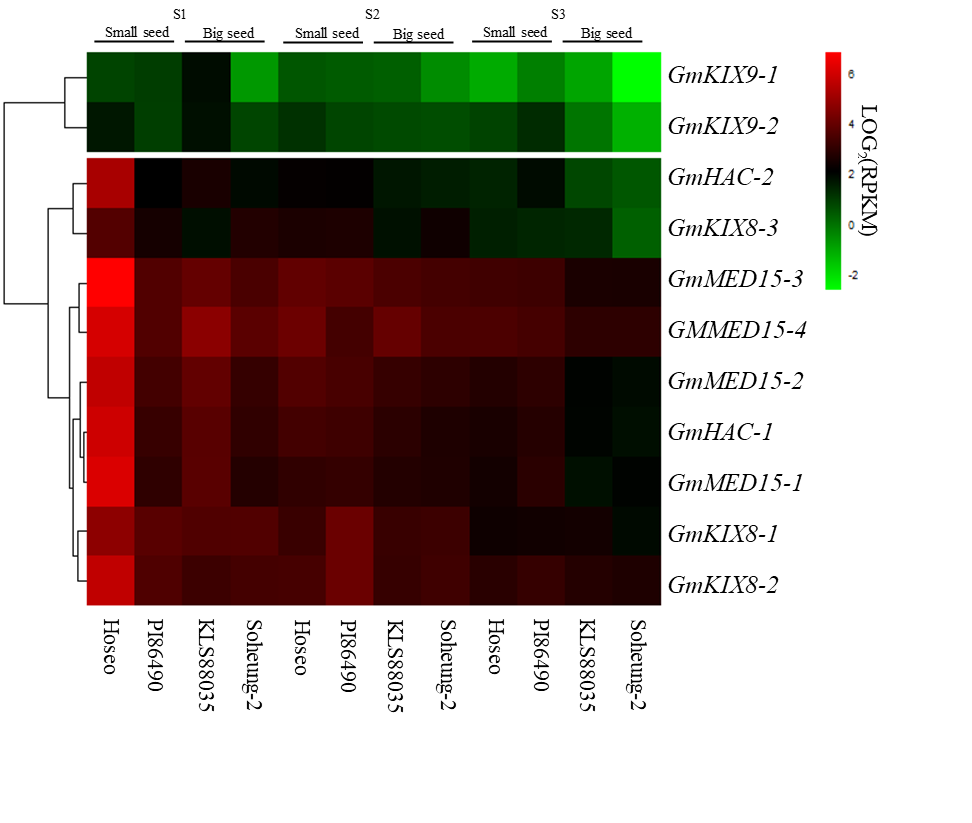


**Supplementary Figure 5**. **Heatmap of 13 *GmKIX* domains expression in three stage of seed development.** The heatmap was Constructed using log_2_ (RPKM) values. The color legend is shown on the right, and gene names are beside the heatmap.
